# Impact of the IL-15 superagonist N-803 on lymphatic reservoirs of HIV

**DOI:** 10.1172/jci.insight.190831

**Published:** 2025-06-03

**Authors:** Joshua Rhein, Jeffrey G. Chipman, Gregory J. Beilman, Ross Cromarty, Kevin Escandón, Jodi Anderson, Garritt Wieking, Jarrett Reichel, Rodolfo Batres, Alexander Khoruts, Christopher M. Basting, Peter Hinderlie, Zachary B. Davis, Anne Eaton, Byron P. Vaughn, Elnaz Eilkhani, Jeffrey T. Safrit, Patrick Soon-Shiong, Jason V. Baker, Nichole R. Klatt, Steven G. Deeks, Jeffrey S. Miller, Timothy W. Schacker

**Affiliations:** 1 Division of Infectious Diseases and International Medicine, Department of Medicine;; 2 Department of Surgery;; 3 Masonic Cancer Center;; 4 Division of Gastroenterology, Department of Medicine;; 5 Division of Hematology, Oncology, and Transplantation, Department of Medicine; and; 6 Division of Biostatistics and Health Data Science, School of Public Health, University of Minnesota, Minneapolis, Minnesota, USA.; 7 Division of HIV, Infectious Diseases and Global Medicine, Department of Medicine, University of California, San Francisco, San Francisco, California, USA.; 8 ImmunityBio, Culver City, California, USA.; 9 Division of Infectious Diseases, Hennepin Healthcare Research Institute, Minneapolis, Minnesota, USA.

**Keywords:** AIDS/HIV, Infectious disease, Immunotherapy, NK cells

## Abstract

**BACKGROUND:**

NK cell function is impaired in people with HIV (PWH), hindering their potential to reduce the lymphoid tissue (LT) reservoir. The IL-15 superagonist N-803 has been shown to enhance NK and T cell function and thus may reduce viral reservoirs.

**METHODS:**

To determine the impact of N-803 on LTs, we conducted a clinical trial where 10 PWH on effective antiretroviral therapy (ART) were given 3 subcutaneous 6 mcg/kg doses of N-803. We obtained PBMCs and lymph node (LN) and gut biopsies at baseline and after the last N-803 dose.

**RESULTS:**

We found a nonstatistically significant approximately 0.50 median log reduction in the frequency of viral RNA^+^ (vRNA^+^) and vDNA^+^ cells/g in the 6 participants with baseline and posttreatment LN biopsies. In the ileum, we observed reductions of vRNA^+^ cells in 8/10 participants and vDNA^+^ cells in all participants. We also found significant inverse correlations between NK cell proliferation and the frequency of vRNA^+^ cells and between NKG2A expression on NK cells and the frequency of vRNA^+^ cells.

**CONCLUSION:**

Our findings suggest N-803 may reduce the HIV reservoir in LTs of PWH on ART, an effect likely mediated by enhanced NK cell function. Controlled studies assessing the impact of NK cell therapy on HIV LTs are needed.

**TRIAL REGISTRATION:**

ClinicalTrials.gov NCT04808908.

**FUNDING:**

NIH grants 5UM1AI126611, UL1TR002494, R01 AI147912, R35 CA283892, and UM1AI164561.

## Abstract 2

In this study with N-803 administered to 10 people with controlled HIV, N-803 reduced the size of the HIV reservoir in lymphoid tissues.

## Introduction

HIV infection has become a chronic manageable condition due to the widespread use of antiretroviral therapy (ART) in people with HIV (PWH), but it is not currently curable because of the persistence of viral reservoirs. During primary infection, HIV establishes tissue reservoirs that include a large population of HIV-infected CD4^+^ T cells with a transcriptionally silent HIV provirus (the “latent” reservoir) (1). These cells are largely maintained via homeostatic proliferation (2, 3), and only a small fraction have an intact provirus that can reactivate from latency (4). Although most studies have focused on circulating CD4^+^ T cells harboring integrated genomes in PWH, the vast majority of these cells reside in tissue sanctuaries such as secondary lymph nodes (LNs) and gut-associated lymphoid tissues (GALTs), even in PWH receiving ART (5–8). Ideally, any strategy to cure HIV would seek to target both the latent reservoir and the reservoir of viral RNA^+^ (vRNA^+^) productively infected cells.

While NK cells are an important part of the innate immune system that target and kill infected cells, their function is frequently impaired in HIV infection (9–12). There are, however, immune-based therapies that are now available with the potential to improve or restore this function (13–15). One such possible intervention is the IL-15 superagonist N-803 (nogapendekin alfa inbakicept, ImmunityBio), which, in animal models of HIV, has been found to activate T cells and NK cells while reversing latency (14, 16–18). In a phase I study of ART-suppressed PWH, we found that N-803 induced virus production; activated CD4^+^ T cells, CD8^+^ T cells, and NK cells; and reduced the size of the reservoir in circulating PBMCs (19).

To assess the ability of N-803 to reduce viral reservoirs, we conducted a phase Ib clinical trial wherein PWH on ART received N-803 and provided both baseline and posttreatment tissue samples, including PBMCs and both LN and gut biopsies. In addition, we investigated NK cell and CD8^+^ T cell function in LNs and GALTs.

## Results

### Protocol.

A phase Ib, single-arm, open-label study (ClinicalTrials.gov NCT04808908) was conducted. Participants were required to be on continuous ART for at least 2 years, to have undetectable plasma HIV RNA measures in the previous 12 months, and to have a screening CD4^+^ T cell count ≥350 cells/μL. Leukaphereses to obtain PBMCs, excisional inguinal LN biopsies, and colonoscopies with ileal biopsies were conducted at baseline (prior to the first dose of N-803) and again 1 week after the third dose of N-803. All 10 participants received 3 s.c. 6 μg/kg doses of N-803 at 21-day intervals. The dose and interval used were informed by our previous dose-escalation trial (19) and studies in people with cancer (20).

### Participants.

**Figure 1:**
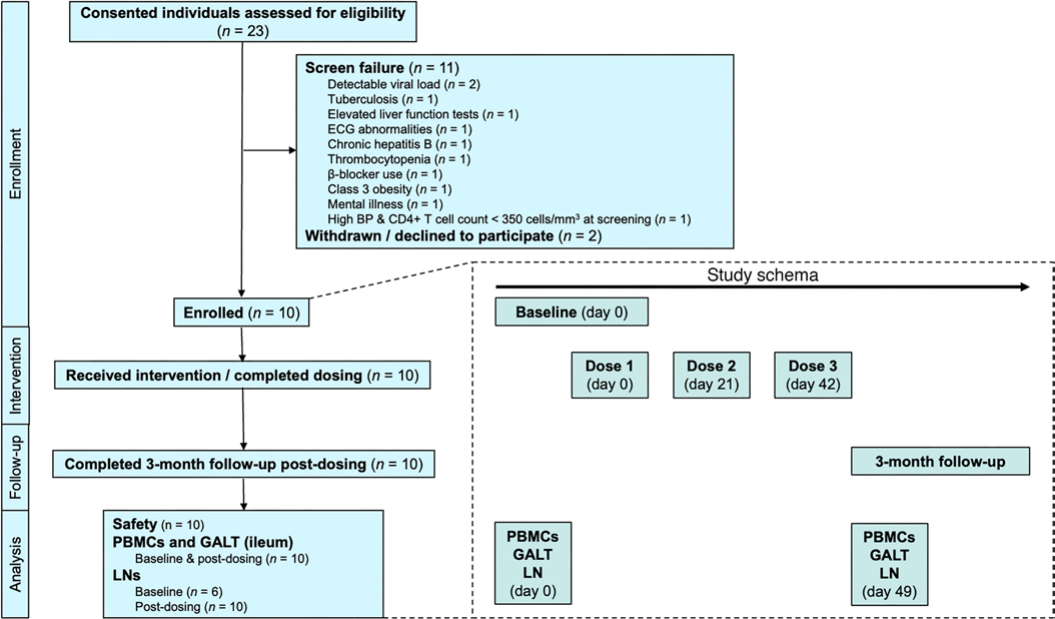
Study schematic. Flow diagram and design of the clinical trial.

We screened and consented a total of 23 individuals (Figure 1), of whom 10 were eligible per our selection criteria and completed dosing. The remaining 13 participants included 11 participants who failed screening criteria and 2 participants who withdrew their consent after successful screening. Primary reasons for screen failure were detectable viral load at screening, evidence of currently active infections such as chronic hepatitis B and tuberculosis, and abnormalities in screening tests conducted per study protocol.

All of the 10 enrolled participants received the planned 3 doses of 6 mcg/kg N-803 by s.c. injection. We performed leukapheresis and colonoscopy with biopsies at both baseline and 1 week after last N-803 dose in all 10 participants. Despite ultrasound guidance, we were unable to locate an LN at baseline in 4 of the individuals; hence, only 6 participants provided pre– and post–N-803 LN samples. Study participants were followed up for 3 months after completing N-803 dosing.

All participants were male, and their median age was 38 years (interquartile range [IQR], 32.3–46.3). Of the 10 participants, 8 identified as White and 2 identified as African American. Ethnicity was non-Hispanic for all participants. The median CD4^+^ T cell count at baseline was 593 cells/μL (IQR 450.3–949.3), and the median CD4/CD8 ratio was 0.87 (IQR 0.56–1.45) (Table 1).

### Impact of N-803 on lymphoid tissue reservoirs of HIV RNA^+^ and DNA^+^ cells.

**Figure 2:**
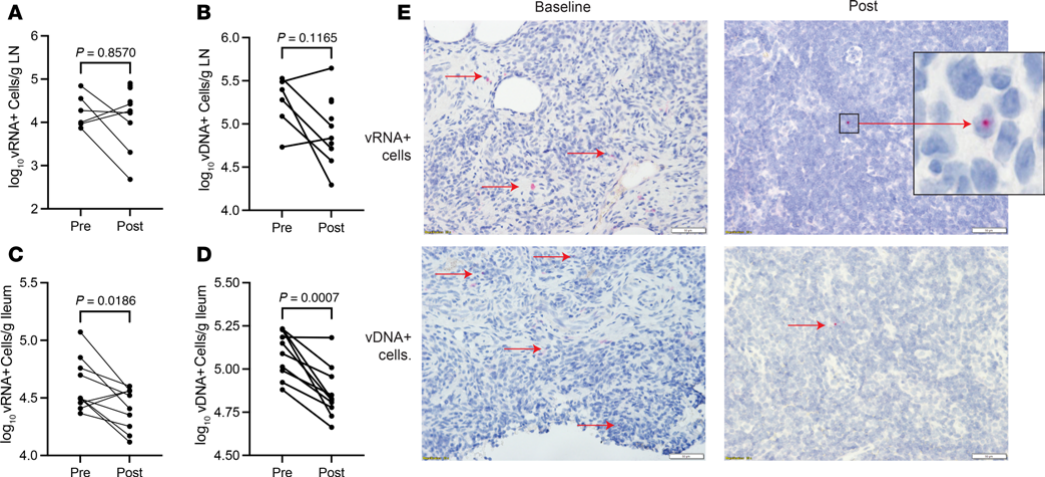
Analysis of changes in frequency of vRNA^+^ and vDNA^+^ cells in LTs. (**A**) Analysis of the frequency of vRNA^+^ cells in the 6 LNs obtained at baseline and the 10 LNs obtained at the post–N-803 time point using HIV RNA in situ hybridization by RNAscope. (**B**) Analysis of the frequency of vDNA^+^ cells by DNAscope. (**C**) Analysis of the frequency of vRNA^+^ cells in the ileum. (**D**) Analysis of the frequency of vDNA^+^ cells in the ileum. (**E**) Representative images from a participant’s LN showing vRNA^+^ and vDNA^+^ cells at the pre– and post–N-803 time points. Scale bar: 50 μm; inset, original magnification, 20×. Statistical analyses performed using an unpaired 2-tailed *t* test for **A** and **B** and paired 2-tailed *t* test for **C** and **D**; *P* values are shown above the pairings.

We first measured the frequency of vRNA^+^ cells in LNs using RNAscope. The median log frequency of vRNA^+^ cells was 4.1 vRNA^+^ cells/g (IQR 3.9–4.6) in the 6 LNs obtained at baseline and 4.3 vRNA^+^ cells/g (IQR 3.8–4.8) in the 10 LNs from the post–N-803 time point (*P* = 0.8570, unpaired 2-tailed *t* test, Figure 2A). For the 6 individuals with LNs obtained at both baseline and post–N-803 time points, there was a median log decrease of 0.46 vRNA^+^ cells/g that was not statistically significant (IQR –0.33–1.20, *P* = 0.21, paired 2-tailed *t* test).

We next measured the frequency of vDNA^+^ cells in LNs using DNAscope (Figure 2B). The median log frequency of vDNA^+^ cells was 5.3 vDNA^+^ cells/g (IQR 5.0–5.5 vDNA^+^) in the 6 LNs obtained at baseline and 4.9 vDNA^+^ cells/g (IQR 4.7–5.3) in the 10 LNs from the post–N-803 time point (*P* = 0.1165, unpaired 2-tailed *t* test). Similar to what we observed for changes in vRNA^+^ cells in paired LNs, there was a median log decrease in the frequency of vDNA^+^ cells of 0.53 cells/g (IQR –0.12–0.70, *P* = 0.085, paired 2-tailed *t* test) that did not reach significance. The change for each of the 6 paired samples was –1.1, –0.56, –0.55, –0.52, +0.10, and +0.16 log vDNA^+^ cells/g LN.

We also measured the frequency of vRNA^+^ and vDNA^+^ cells/g in ileum at both time points for all 10 participants and found a modest decline in the frequency of vRNA^+^ cells in 8/10 participants (Figure 2C). The median change was –0.15 log (IQR –0.50–0.15 log). While this decline was small and of unknown biological significance, the differences were statistically significant (*P* = 0.0186, paired 2-tailed *t* test). There was also a decrease in the frequency of vDNA^+^ cells/g in all 10 participants in the ileum (Figure 2D); the median change was –0.21 log (IQR –0.003 to –0.5 log, *P* = 0.0007, paired 2-tailed *t* test). Figure 2E shows representative images of a participant’s LN analyzed for vRNA^+^ and vDNA^+^ cells at the pre– and post–N-803 time points.

To ensure that changes to vRNA^+^ and vDNA^+^ cell populations were not the result of a “dilutional effect” secondary to cells trafficking in and out of the LNs, we used quantitative image analysis (QIA) to calculate the frequency of CD4^+^ T cells in the T cell zone (TZ) of the paired LNs from 5 of the participants. Because the vRNA^+^ and vDNA^+^ measurements were on CD4^+^ T cells, we reasoned that a dilutional effect would be reflected by changes in the size of the CD4^+^ T cell population in the TZ. We found the median percentage change between time points was 0.6% (range 0.22%–0.87%); thus, any change in the size of the reservoir was not due to a dilutional effect from cells moving in and out of the LNs.

### Measures and function of CD8^+^ T cells and NK cells.

**Figure 3:**
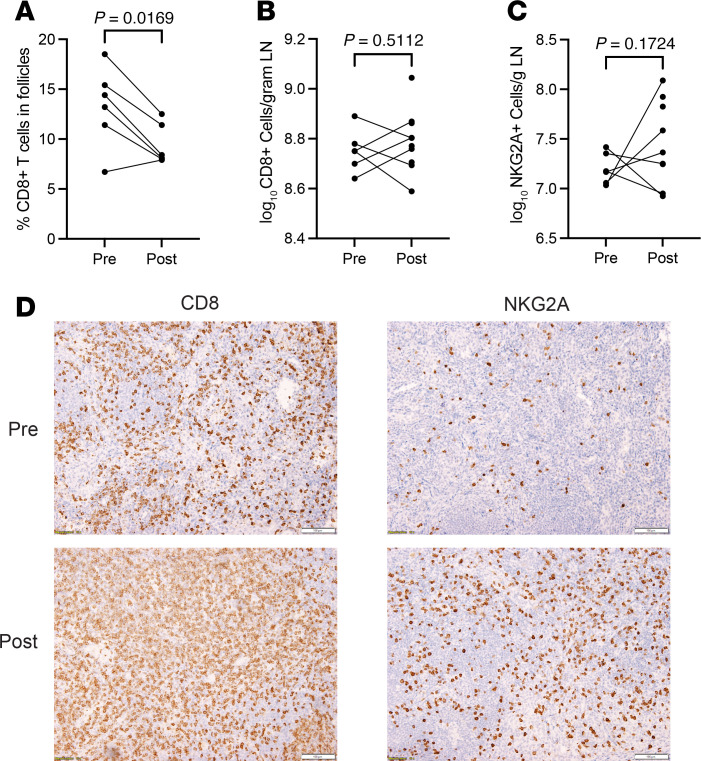
Measures and function of CD8^+^ T cells and NK cells in LNs. (**A**) Frequency of CD8^+^ T cells in B cell follicles in the 6 individuals with both a pre– and post–time point. We counted CD8^+^ T cells inside of B cell follicles and in the parafollicular T cell zone to provide an estimate of the percentage of those cells in the B cell. (**B**) Grouped analysis of the log frequency of CD8^+^ T cells in the group with 6 baseline LN samples and the group with 10 post–N-803 LN samples. (**C**) Grouped analysis of the log frequency of NKG2A^+^ cells in 6 pre– and 10 post–N-803 LNs. (**D**) Representative images from a participant’s LN with an increase in both CD8^+^ and NKG2A^+^ cells at the pre– and post–N-803 time points. Scale bar: 100 μm. Statistical analyses performed using a paired 2-tailed *t* test in **A** and an unpaired 2-tailed *t* test in **B** and **C**; *P* values shown above pairings.

We next examined the impact of N-803 on CD8^+^ T cell and NK cell populations in LNs and GALTs, as N-803 is known to cause activation and proliferation of these effector cells. We stained tissues with antibodies directed against CD8^+^ T cells and used QIA to measure the frequency of CD8^+^ T cells in the parafollicular TZ and B cell follicles (identified by double-label staining with CD20 to mark B cells). We were particularly interested in the changes in these populations by anatomic location in the LNs because of a report of an increase in SIV-specific CD8^+^ T cells in follicles after N-803 treatment in a nonhuman primate (NHP) model of SIV infection (21). In contrast with that study, we found CD8^+^ T cells significantly decreased in follicles after N-803 therapy (*P* = 0.0169, paired 2-tailed *t* test, Figure 3A). However, when analyzing the whole tissue section, we detected no significant change in the frequency in LNs overall (Figure 3B, *P* = 0.5112, unpaired 2-tailed *t* test), with some individuals having an increase and some a decrease.

The NK cell population was examined by using antibodies directed against NKG2A to detect NK cells in the LTs. We recognize that a very small population of T cells can express the NKG2A receptor, but for purposes of this histologic analysis (where double-label image analysis was not feasible), we restricted our analyses to using only the NKG2A marker. Further, NK cells in LNs are mostly of the CD56^bright^ phenotype, which highly express NKG2A (22). There was no significant change in the overall frequency of NK cells in LNs (Figure 3C, *P* = 0.1724, unpaired 2-tailed *t* test). Among the paired samples (6 participants), there were 3 with a log increase of 1.06, 0.53, and 0.19 NK cells and 3 with a log decrease of 0.49, 0.22, and 0.10 NK cells. In addition, there was no change in the frequency of NK cells in the ileum (*P* = 0.94, paired 2-tailed *t* test, Supplemental Figure 1; supplemental material available online with this article; https://doi.org/10.1172/jci.insight.190831DS1). Figure 3D shows representative images of a participant’s LN analyzed for CD8^+^ and NKG2A^+^ cells at the pre– and post–N-803 time points.

**Figure 4:**
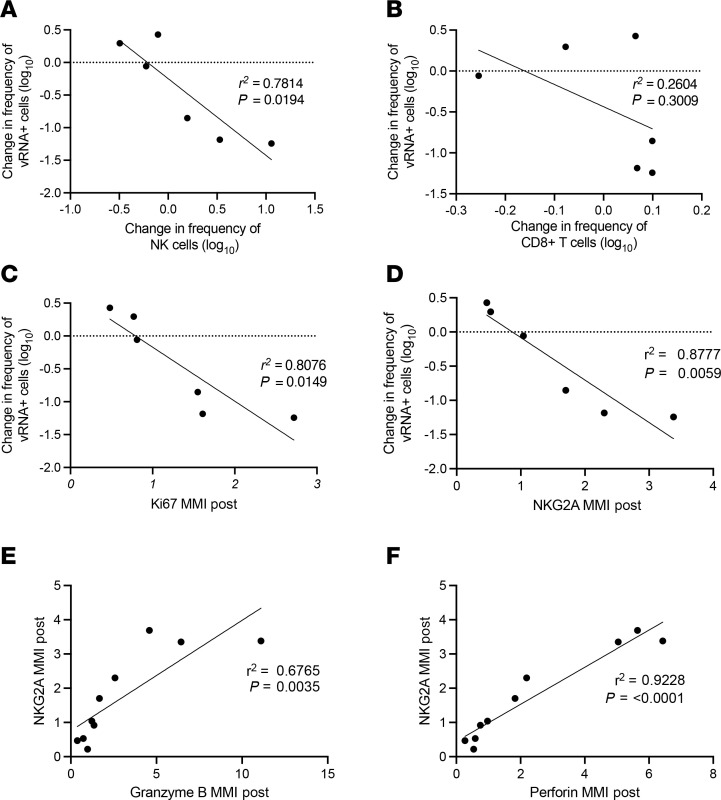
Correlations between vRNA^+^ frequency, cell types, and immune markers in LN samples. (**A**) Correlation between the frequency of vRNA^+^ cells and the frequency of NK cells measured by ISH and IHC, respectively. (**B**) Correlation between the frequency of vRNA^+^ cells and the frequency of CD8^+^ T cells measured by ISH and IHC, respectively. (**C**) Correlation between the frequency of vRNA^+^ cells and Ki-67 expression measured by ISH and CyTOF, respectively. (**D**) Correlation between the frequency of vRNA^+^ cells and NKG2A expression measured by ISH and CyTOF, respectively. (**E**) Correlation between NKG2A expression and granzyme B expression measured by CyTOF. (**F**) Correlation between NKG2A expression and perforin expression measured by CyTOF. Analyses performed by linear regression; *P* values displayed on each panel. MMI, mean metal intensity.

We were interested to know the functional capacity of NK cells and CD8^+^ T cells that were activated by N-803. We performed cytometry by time-of-flight (CyTOF) analyses on LN mononuclear cells (LNMCs) from the 6 individuals with paired LN samples to characterize N-803–associated changes to proliferation (Ki-67) and cytolytic activity (granzyme B and perforin expression). While we did not find any consistent change in markers of proliferation or cytolytic activity (Supplemental Figure 2), expression of these markers can be transient and, given that we obtained the postintervention LNs 1 week after the last N-803 dose, changes could have occurred. We postulated that if either effector cell population had changes to cytolytic activity, there might be a relationship between the change in frequency of vRNA^+^ cells and the change in frequency of the effector cell population. When we compared the change in frequency of vRNA^+^ cells in LNs with changes in the frequency of NK cells or CD8^+^ T cells (Figure 4, A and B), we found a significant inverse correlation for NK cells (*r*^2^ = 0.7814, *P* = 0.0194) by linear regression but not for CD8^+^ T cells (*r*^2^ = 0.2604, *P* = 0.3009). Thus, the individuals with the greatest increase in NK cells, but not CD8^+^ T cells, had the greatest reduction in frequency of vRNA^+^ cells in LNs.

We also examined the relationship between expression of Ki-67 in NK cells as a marker of cell proliferation and the reduction in frequency of vRNA^+^ cells in LNs and found a significant inverse correlation between NK cell proliferation and reservoir reduction (Figure 4C, r^2^ = 0.8076, *P* = 0.0149, linear regression). In addition, NK cells are known to upregulate NKG2A expression in response to activation as a mechanism to limit aberrant NK cell activation and cytolytic function (23, 24). We found a significant inverse relationship between increased expression of NKG2A on NK cells and a decrease in the frequency of vRNA^+^ cells (Figure 4D, r^2^ = 0.8777, *P* = 0.0059, linear regression).

Finally, we analyzed the relationship between expression of NKG2A and both granzyme B and perforin in the 10 LNs obtained at the post–N-803 time point to further characterize the functional capacity of NK cells and found that increased expression of NKG2A was associated with increased expression of both granzyme B (Figure 4E, r^2^ = 0.6765, *P* = 0.0035, linear regression) and perforin (Figure 4F, r^2^ = 0.9228, *P* < 0.0001, linear regression), which would be expected to enhance effector function. These correlations support a model where improved function of NK cells leads to reductions in viral reservoirs. However, mechanistic studies will need to be done.

### Safety of N-803.

Similar to what we reported in our dose-escalation trial (19), there were no unexpected safety signals, and most adverse events (AEs) were grade 1 (mild). Plasma HIV RNA remained undetectable in all participants through study completion. All participants had grade 1 AEs, 7/10 participants had grade 2 AEs, and 8/10 participants had grade 3 AEs. The grade 3 AEs were primarily injection site reactions in 8 participants. We note that, per the arrangement made with the FDA during the previous dose-escalation study (19) and with institutional review board (IRB) approval, grade 3 injection site reactions including erythema and induration were deemed expected and not considered clinically significant toxicities. In addition, there were 4 episodes of low estimated glomerular filtration rate (eGFR) in 2 individuals, including 3 reports in 1 participant whose screening eGFR was 55 mL/min (grade 3 per the Division of AIDS AE table) and an isolated report at day 7 in another individual who had otherwise-normal creatinine clearance calculations throughout the study. We provide detailed assessments of clinical and laboratory AEs in Supplemental Tables 1 and 2, respectively.

## Discussion

In this open-label trial of N-803 in ART-suppressed PWH, we found evidence for a reduction of both vRNA^+^ and vDNA^+^ cells in LNs and ileum following N-803 therapy. There was a median 0.46 log reduction in the frequency of vRNA^+^ cells/g and a median 0.53 log reduction in the frequency of vDNA^+^ cells/g in participants with paired LNs, though these did not reach statistical significance. In the ileum samples, we found a small but statistically significant reduction of vRNA^+^ and vDNA^+^ cells. These reductions were observed after only 3 doses of N-803 administered over a 6-week interval. It is possible that a longer duration of therapy would result in larger reductions in the size of these viral reservoirs. Determination of effects of extended dosing awaits additional clinical study.

These results were not anticipated, particularly for the change in frequency of vDNA^+^ cells. However, this was a small number of participants, and the results were not statistically significant. Further studies are needed in a larger sample size to verify this observation. We point out that there are relatively few studies that have looked at the impact of an intervention like N-803 in LNs. More studies with NK cell–based therapies are warranted given that NK cells recognize infected cells through multiple pathways, including detection of cell stress ligands expressed on the infected cell surface, absence of MHC-I molecules, and antibody binding to the infected cell (25). They do not necessarily require expression of viral proteins to kill an infected cell.

In previous clinical trials using N-803, there was marked proliferation and activation of both CD8^+^ T cells and NK cells in the peripheral blood. In this study, we did not detect significant changes in the CD8^+^ T cell or NK cell populations in LTs (Figure 3). In contrast with our original hypothesis, we observed a significant reduction of CD8^+^ T cells in B cell follicles. This finding contrasts with an NHP study, wherein N-803 increased the frequency of SIV-specific CD8^+^ T cells in B cell follicles and decreased the frequency of vRNA^+^ T follicular helper cells (21). Possible explanations for these disparate outcomes, beyond important species differences, are the dosing and administration of the drug. In the present clinical study, 3 doses of N-803 at 6 mcg/kg were administered s.c., whereas in the SIV study, intravenous bolus doses of either 6 mcg/kg or 100 mcg/kg were given.

Mechanistically, we found evidence that N-803–mediated increases in NK cell function were associated with a reduction in the reservoir tissue size. When we compared changes in the frequency of vRNA^+^ cells with changes in the frequency of NK cells and CD8^+^ T cells, we found a significant correlation with NK cells but not CD8^+^ T cells. In addition, we found a significant correlation between reductions in vRNA^+^ cells and expression of the NK cell marker NKG2A (Figure 4).

NKG2A is an inhibitory receptor that downregulates NK cell function after contact with its ligand HLA-E. Yet, NKG2A is also upregulated in tissues and by activation with γ cytokines such as IL-2 or IL-15. This suggests that HLA-E suppression by infected T cells is not a dominant mechanism to suppress NK cells (25), but is rather a marker of NK cell activation as we show here, allowing NK cells expressing NKG2A to still degranulate and perform cytolytic functions (26).

This study has several limitations. This is a small, single-arm study with no control arm. In addition, we were not able to conduct additional experiments to verify our in situ findings of reductions in the vRNA^+^ and vDNA^+^ reservoir. This was due to the average LNMC yield from disaggregation of an LN being approximately 5 × 10^6^ cells, insufficient for most molecular assays measuring viral reservoirs, which require at least 10^7^ LNMCs. Reasons for a smaller cell yield include the reduced T cell populations in LNs of PWH (27–29) and our dissection of the LN into 2 parts so that a half could be used for histologic analyses. Despite these limitations, there are data to show that in situ measures of vRNA^+^ cells are as sensitive as molecular methods (30). A further limitation was the lack of diversity of study participants, who were all male, with 8 being White. This is relevant given that immune function is affected by biological sex and perhaps other factors, such as race. Future studies are needed with larger numbers of diverse participants to address these limitations.

Despite the study limitations, we were able to show a potential impact of N-803 therapy on viral reservoirs in LTs and that NK cells are the likely mechanism of reduction. N-803, as NK cell–directed therapy, could be part of an overall HIV cure strategy that may also comprise broadly neutralizing antibodies, latency-reversing agents such as HODHBt, or haploidentical donor NK or other adoptive NK cell therapy (31–33). Establishment of an optimal multimodal therapy for HIV that could contribute to a cure awaits future clinical study.

## Methods

### Sex as a biological variable.

Sex was considered as a biological variable. Our protocol was written to include any eligible participant regardless of sex, ethnicity, or race, and our recruitment efforts reflected that principle. The cohort enrolled in this study generally reflected the demographic characteristics of PWH in Minnesota. All study participants were male.

### Study design and population.

The study was conducted at the University of Minnesota (UMN). The first participant was enrolled on March 3, 2021, and the last participant on December 22, 2021. Inclusion criteria included ages 18–65 years, stable ART for over 2 years with no interruptions greater than 14 consecutive days and no plans to modify ART during the study period, undetectable plasma HIV RNA measures at screening and in the previous year (HIV RNA < 20 copies/mL; isolated single blips between 20 and 200 copies/mL were allowed if preceded and followed by undetectable viral load determinations), and a screening CD4^+^ T cell count ≥350 cells/μL (Supplemental Methods). Exclusion criteria included other active infections (e.g., hepatitis B and C, tuberculosis); pregnancy or breastfeeding; clinically significant cardiovascular, pulmonary, renal, hepatic, neurological, gastrointestinal, or psychiatric/mental diseases or disorders; active or recent malignancy requiring systemic chemotherapy or surgery in the preceding 3 years; treatment with or exposure to oral or systemic immunomodulatory drugs in the 30 days prior to screening; and marked abnormalities in electrocardiogram or pulmonary function test. Most screening laboratory tests needed to be within laboratory normal range and not higher than grade 1 as defined by the NIH Division of AIDS Table for Grading the Severity of Adult and Pediatric AEs, Corrected Version 2.1 (https://rsc.niaid.nih.gov/clinical-research-sites/daids-adverse-event-grading-tables), with the exception of the eGFR, which needed to be 50 mL/min, as PWH often have baseline renal function abnormalities.

### Study activities and procedures.

Participants were recruited using IRB-approved online and print advertisements and were also referred from local clinics. N-803 was administered to participants at 6 μg/kg for 3 s.c. doses in the abdomen at day 0, day 21, and day 42 at the UMN Phase 1 Clinical Research Unit (CRU) of the UMN Minneapolis campus (Figure 1). The dose and the 3-week interval of administration were determined by our previous dose-escalation study (19); weight was capped at 100 kg for dose calculations. Participants were observed in the CRU for a minimum of 2 hours after each outpatient dose. Acetaminophen and diphenhydramine were administered approximately 30 minutes prior to each dose to every participant per study protocol. Participants were also instructed to take another dose of these medications 4 hours after the time of study drug administration. Leukaphereses were carried out at the Apheresis Unit of the MHealth Clinics and Surgeries Center on the Minneapolis campus of the UMN using institutional standard procedures. Colonoscopies were conducted at the Endoscopy Center of the UMN Medical Center at the UMN Minneapolis campus under moderate sedation and after overnight bowel preparation. Pinch biopsies were done in the terminal ileum (Radial Jaw 4 Biopsy Forceps, Jumbo with Needle, 3.2 mm; Boston Scientific). Excisional inguinal LN biopsies were completed by experienced surgeons at the CRU under ultrasound guidance and with administration of local anesthesia. All study visits, involving study activities such as vitals, physical exams, blood draws, follow-ups, and research data collection, occurred in the CRU. Demographics, medical history, concomitant medications, AEs, and clinical data were all collected in REDCap.

### Clinical trial oversight.

The protocol was administered under the FDA investigational new drug application 125191. The protocol was detailed in that we were collecting all safety information and had clear stopping rules and pause criteria depending on the safety information collected. A Safety Monitoring Committee (SMC) was convened to provide trial oversight. This committee was composed of 3 independent scientists selected based on their expertise in the clinical management of HIV infection or the conduct of clinical trials. The SMC reviewed clinical data after the first participant was enrolled and then biannually. It was also responsible for reviewing all grade 3 toxicities and making a formal recommendation about pausing or continuing with the study. All AEs graded 3 and any related, unexpected, and serious AEs were reported to the SMC. All SMC reports were provided to the UMN IRB and the FDA as required. The NIH Division of AIDS Table for Grading the Severity of Adult and Pediatric AEs (corrected version 2.1 from July 2017) was used to assess and grade clinical and laboratory AEs. The relationship of clinical AEs to study drugs and procedures was assessed by the study investigators.

### Endpoints.

The primary endpoints were 1) to determine the safety of N-803 given at this dose and frequency in this population and 2) to determine the frequency of CD8^+^ T cells in follicles before and after N-803 therapy. The secondary endpoint was the change in the frequency, location, and phenotype of vRNA^+^ and vDNA^+^ cells in LTs. Several exploratory endpoints were planned, including the frequency, the location, and the function of CD8^+^ T cells and NK cells.

### Sample size.

Because this study was the first of its kind to examine the impact of N-803 on CD8^+^ T cells in B cell follicles in humans, preliminary data were limited. In the study by Webb et al. (21), in 9 tissue samples from 6 SIV-infected macaques, the frequency of SIV-specific CD8^+^ T cells/mm^2^ in the B cell follicle drastically increased after N-803 administration. The average change divided by the standard deviation was approximately 1.11, resulting in a paired, 2-sided *t* test *P* value of 0.0102. Assuming N-803 had a similar response in humans (Cohen’s *d* = 1.11) and using a type I error rate of 0.05, our study with 10 participants would have approximately 87% power to detect an effect of this size or greater.

### Clinical laboratory.

Blood CD4^+^ T cell counts were measured by flow cytometry using FACSCount (Becton Dickinson Inc). Plasma HIV viral load was measured using the COBAS AmpliPrep/COBAS TaqMan 96 (Roche). Both platforms were registered on an external quality assurance program provided by the American Pathologists and Virology Quality Assurance from Rush University Medical Center.

### Cell separation of PBMCs.

Blood was collected in acid citrate dextrose solution A (ACD-A) tubes (Becton Dickinson Inc) tubes. Tubes were spun at 400*g* for 10 minutes with the brake off. During this spin, 13 mL of Histopaque-1077 (MilliporeSigma) was added to each of the SepMate 50 mL tubes (STEMCELL Technologies). The plasma was removed from the spun ACD-A tubes and frozen in 1 mL aliquots. The plasma was replaced with 1× PBS and transferred to 50 mL tubes, where the volume was adjusted to 32.5 mL. The blood/PBS mixture was pipetted into the prepared SepMate tubes. The tubes were then centrifuged at 1,200*g* for 10 minutes at room temperature with a brake. The buffy coat layer was then poured off and divided between two 50 mL conical tubes. Complete RPMI (CRPMI) was added to each tube to bring total volume to 50 mL. Tubes were centrifuged at 400*g* for 10 minutes at room temperature and supernatant was discarded. Pellets were combined and resuspended in 25 mL of CRPMI and then centrifuged at 250*g* for 10 minutes to remove platelets. The pellet was resuspended in 20 mL of CRPMI. A 10 μL aliquot was removed and added to 90 μL of trypan blue for counting by hemocytometer. The cell suspension was centrifuged at 400*g* for 5 minutes at room temperature. The cell pellet was then resuspended in Freezing Media (90% FBS:10% DMSO) to achieve a concentration of 1 × 10^7^ cells/mL.

### Peripheral blood CD4^+^ T cell, CD8^+^ T cell, and NK cell counts.

Absolute CD4^+^ T cells, CD8^+^ T cells, and NK cells were determined by multiplying the absolute lymphocyte count from the research complete blood count by the percentage of the flow cytometry lymphocyte gate containing CD56^+^CD3^–^ NK cells, CD3^+^CD4^+^ T cells, and CD3^+^CD8^+^ T cells.

### ISH.

These methods have been previously described (5, 34). Five to ten 5 μm sections separated by 20 μm were analyzed by RNAscope 2.5 (ACD). The anti-sense (for the detection of vRNA) HIV probes used cover approximately 4.5 kb of the genome and are designed to bind to sequences in *gag*, *pol*, *vif*, *vpx* (for SIV), *vpr*, *tat*, *rev*, *vpu* (for HIV), *env*, and *nef*. HIV RNA–specific probes from Advanced Cell Diagnostics were used for HIV RNA ISH to identify Clade B viruses (catalog number 416111 and DNA sense catalog number 425531).

### Immunohistochemistry.

Five μm–thick sections of paraffin-embedded tissue mounted on glass, positively charged slides (Creative Waste Solutions) were used for all staining protocols. The tissues were deparaffinized in xylenes followed by graded ethanol and hydrated in deionized water. After antigen retrieval, the tissues were blocked with Sniper (Biocare Medical) for 30 minutes. Primary antibodies NKG2A (Abcam), CD8 (Thermo Fisher Scientific), or CD20 (Biocare) were added overnight at 4°C. Secondary HRP antibodies (GBI) were added after washing 3 times in Tris-buffered saline with Tween. DAB (Vector Laboratories) was added per instructions and counterstained with CAT Hematoxylin (Biocare Medical). Following counterstaining, tissues were dehydrated and mounted in Permount, ready to be imaged on the Aperio Versa 8 whole slide imaging scanner (Leica Biosystems). A list of the antibodies with a description or reference to the source, catalog, and clone number, where applicable, is provided in Supplemental Table 3.

### QIA.

Photographic images were captured and the frequency of vRNA^+^ cells were measured and expressed as the total per unit area. These methods have been extensively reviewed (27, 35, 36). Briefly, the area of the tissue is measured using image analysis software and expressed as μm^2^. The tissue is 5 μm thick, and by knowing the area of tissue in μm^3^, we then convert that to cm^3^. The average density of LT is 1 g/cm^3^ (37), providing a measure of cells/g.

### Barcoding and staining for CyTOF.

PBMCs were thawed and counted, and viability was measured using trypan blue exclusion on the Countess II FL Automated Cell Counter (Thermo Fischer Scientific). A total of 500,000 viable cells were plated in a 96–deep well plate and rested overnight in R10 media, which is composed of RPMI 1640 (Corning, 10-041-CV), 10% fetal bovine serum (Cytiva, SH30088.03HI), and 1% Penicillin/Streptomycin (Gibco, 15140-122). The next morning, cells were pelleted by centrifugation at approximately 400*g* and barcoded using the Cell-ID 20-Plex Pd Barcoding Kit (Standard Biotools Product 201060) as per manufacturer’s instructions. Briefly, cells were washed and stained with cisplatin (Standard Biotools Product 201064), fixed, permeabilized, and then incubated with the appropriate barcodes for 30 minutes, with a gentle mix after 15 minutes. Following incubation, cells were washed, and all 20 barcoded samples were combined into a single 5 mL, polystyrene, U-bottom tube for staining. This combined sample was stained with the surface marker antibody cocktail for 30 minutes at 4°C. After surface staining, cells were permeabilized by incubation with Triton X 0.1% for 5 minutes at room temperature, followed by incubation with intracellular antibody cocktail for 30 minutes at 4°C. Following intracellular stain, cells were washed twice and then fixed with 2% paraformaldehyde for 10 minutes at room temperature. Stained cells were then incubated at room temperature for 1 hour with Cell-ID Intercalator (Standard Biotools Product 201192A). Wash steps were completed using either Maxpar PBS during barcoding (Standard Biotools Product 201058) or Maxpar Cell Staining Buffer during staining (Standard Biotools Product 201068) at 1,600 rpm for 6 minutes unless stated otherwise. Samples were washed and kept pelleted overnight in Millipore water (MilliporeSigma) and run on the Helios Mass Cytometer (Standard Biotools). Samples were debarcoded on the Helios machine using the CyTOF software v7.0.8493 and exported as individual FCS files for further analysis using the FlowJo version 10.8.1 software (BD Life Sciences). A list of the antibodies with a description or reference to the source, catalog, and clone number, where applicable, is provided in Supplemental Table 3.

### Tagging antibodies with metal labels.

Conjugation of heavy metals to a specific ScFv was conducted using the Maxpar antibody labeling kit (Standard Biotools). The protocol involves partial antibody reduction using 0.5 M TCEP: Pierce Bond-Breaker TCEP Solution (Thermo Fisher Scientific 77720), as well as comprehensive buffer exchange using centrifugal filter units of both 3 kDa and 50 kDa size (MilliporeSigma UFC500396, UFC505096). After conjugation of the antibody, yield is measured, and the final reagent is stored in antibody stabilizer (Boca Scientific 131 000). The reagent is then titrated and verified against known flow cytometry antibodies (38–40).

### CyTOF analyses.

Individual debarcoded FCS files were analyzed using FlowJo version 10.8.1 software. NK cells were gated by expression of CD56 or CD16 after exclusion of dead cells and monocytes, B cells, and T cells (Supplemental Figure 3). In Supplemental Figure 4, we show a comparison between CyTOF staining and flow cytometry staining of NK cells. HIV infection is associated with the loss of CD56 expression on NK cells (41, 42). Therefore, CD56^–^CD16^+^ cells were also included as NK cells, in addition to traditional CD56^+^CD16^+/–^ NK cells. Due to the low number of events acquired in CyTOF, we used the geometric mean for each one of the markers of the total population of NK cells and CD8^+^ and CD4^+^ T cells. This strategy enables assessment of changes between groups of the total population of cells. The geometric mean metal intensity of individual markers was exported as an Excel file, and data were extracted into Prism v10 (GraphPad) for further data analysis. To identify statistical differences between time points in the proliferative and functional capacity of NK cells and CD8^+^ T cells in LNs, a paired 2-tailed *t* test was performed.

### Statistics.

Statistical analyses were performed using Prism (GraphPad Software). Descriptive analyses included the median and IQR for numerical variables. We used a paired 2-tailed *t* test to compare the frequency of immune cells before and after the intervention (N-803) for participants with paired samples. As detailed in the *CyTOF analyses* section, a grouped mixed effects analysis with Holm-Šidák multiple comparisons test was performed when comparing geometric mean immune marker levels between different groups at the same time. The Pearson’s correlation coefficient (Pearson’s *r*) was used to measure the correlation between the frequency of vRNA^+^ cells and immune cells (NK and CD8^+^ T cells) after log_10_ transformation of the data. *P* values less than 0.05 were considered statistically significant.

### Study approval.

The UMN IRB approved the study (UMN IRB STUDY0007810). All participants gave written informed consent using IRB-approved forms. The trial was registered on ClinicalTrials.gov (NCT04808908) in March 2021.

### Data availability.

Data values for all figures can be found in the Supporting Data Values file.

## Author contributions

All authors made substantial contributions to this work. J Rhein provided oversight for the clinical components of the trial at UMN. JGC and GJB performed the research LN biopsies. JA, RC, KE, GW, J Reichel, JSM, PH, ZBD, CMB, and NRK provided laboratory or statistical data analyses. RB and KE oversaw administrative details and implementation aspects of the trial and contributed to identifying participants and following them through the clinical protocol. AE provided statistical guidance. AK and BPV performed the research colonoscopies. JTS and PSS provided N-803 for the trial but had no influence on study design, execution of the clinical trial, or data interpretation. JVB provided oversight for the conduct of the trial at Hennepin Healthcare Research Institute. SGD and EE assisted with administrative and regulatory support. JSM, SGD, and TWS designed the clinical protocol and provided oversight for the conduct of the trial. TWS, JA, and KE wrote the manuscript.

## Supplementary Material

Supplemental data

ICMJE disclosure forms

Supporting data values

## Figures and Tables

**Table 1:**
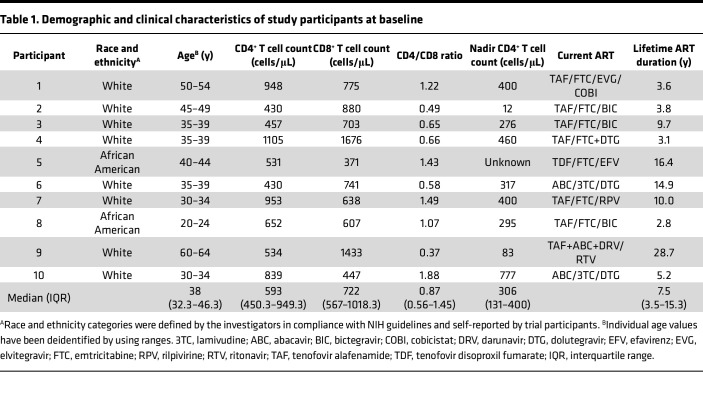
Demographic and clinical characteristics of study participants at baseline

## Version 1 06/05/2025

In-Press Preview

## Version 2 07/08/2025

Electronic publication
