# Supplementary material for: Impact of the IL-15 superagonist N-803 on lymphatic reservoirs of HIV
Source: JCI Insight. 2025 Jun 3;10(13):e190831. doi: 10.1172/jci.insight.190831 (PMC12288898; doi:10.1172/jci.insight.190831)
Supplement: Supplemental data [file jciinsight-10-190831-s081.pdf]

SUPPLEMENTAL FIGURES, TABLES, AND METHODS

Impact of the IL-15 superagonist N-803 on lymphatic reservoirs of HIV

Joshua Rhein[1], Jeffrey G. Chipman[2], Gregory J. Beilman[2], Ross Cromarty[3], Kevin Escandón[1], Jodi Anderson[1], Garritt Wieking[1], Jarrett Reichel[1], Rodolfo Batres[1], Alexander Khoruts[4], Christopher M. Basting[2], Peter Hinderlie[5], Zachary B. Davis[5], Anne Eaton[6], Byron P. Vaughn[4], Elnaz Eilkhani[7], Jeffrey T. Safrit[8], Patrick Soon-Shiong[8], Jason V. Baker[1,9], Nichole R. Klatt[2], Steven G. Deeks[7], Jeffrey S. Miller[3,5], and Timothy W. Schacker[1]

[1]Division of Infectious Diseases and International Medicine, Department of Medicine, University of Minnesota, Minneapolis, MN, USA
[2]Department of Surgery, University of Minnesota, Minneapolis, MN, USA
[3]Masonic Cancer Center, University of Minnesota, Minneapolis, MN, USA
[4]Division of Gastroenterology, Department of Medicine, University of Minnesota, Minneapolis, MN, USA
[5]Division of Hematology, Oncology, and Transplantation, Department of Medicine, University of Minnesota, Minneapolis, MN, USA
[6]Division of Biostatistics and Health Data Science, School of Public Health, University of Minnesota, Minneapolis, MN, USA
[7]Division of HIV, Infectious Diseases and Global Medicine, Department of Medicine, University of California, San Francisco, San Francisco, CA, USA
[8]ImmunityBio, Culver City, CA, USA
[9]Division of Infectious Diseases, Hennepin Healthcare Research Institute, Minneapolis, Minnesota, USA

Address correspondence to: Timothy W. Schacker, University of Minnesota, MMC 250, 420 Delaware Street SE, Minneapolis, Minnesota 55455, USA. Phone: 612-624-9955; Email: schacker@umn.edu.

Supplemental Figure 1. Measures of CD8+ T cells and NK cells in the ileum. (A) Grouped analysis of the log frequency of CD8+ T cells in the 10 baseline ileum samples and 10 post-N-803 ileum samples. **(B)** Grouped analysis of the log frequency of NK cells in the 10 baseline ileum samples and 10 post-N-803 ileum samples. *P* values were determined by a paired *t* test.

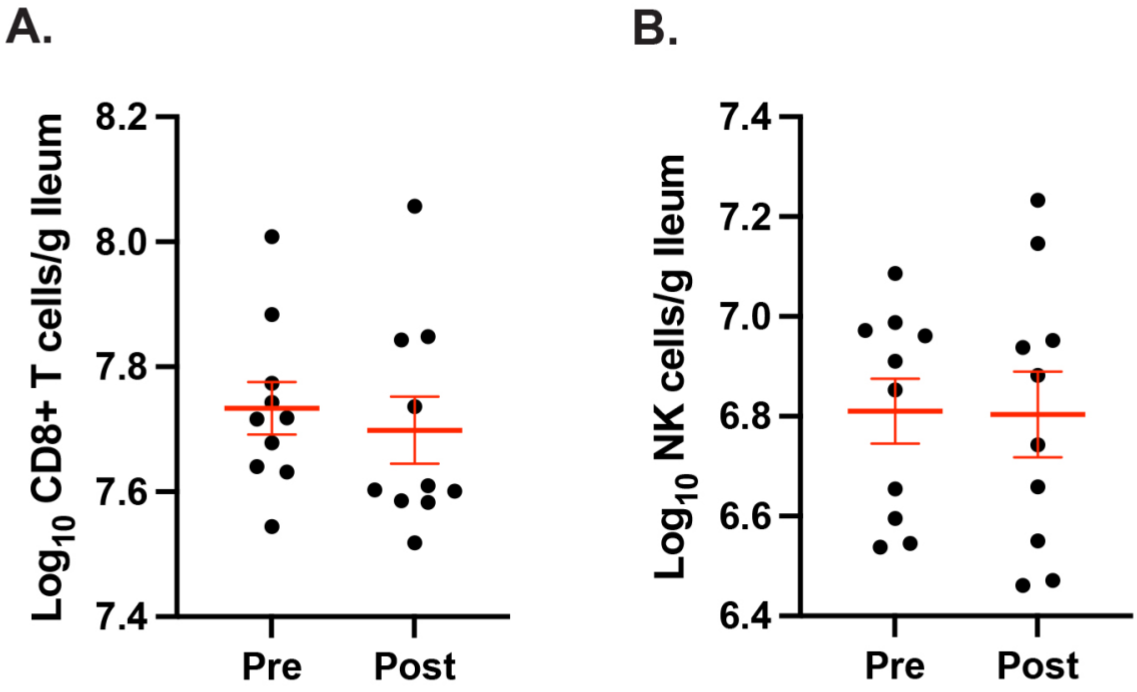

Supplemental Figure 2. Paired analysis of Mean Metal Intensity (MMI) by CyTOF for Ki-67, perforin, and granzyme B expression in CD8+ T cells and NK cells. Samples are from the baseline and post-N-803 timepoints of the 6 individuals for whom we obtained paired lymph nodes. All *P* values were determined by a paired *t* test.

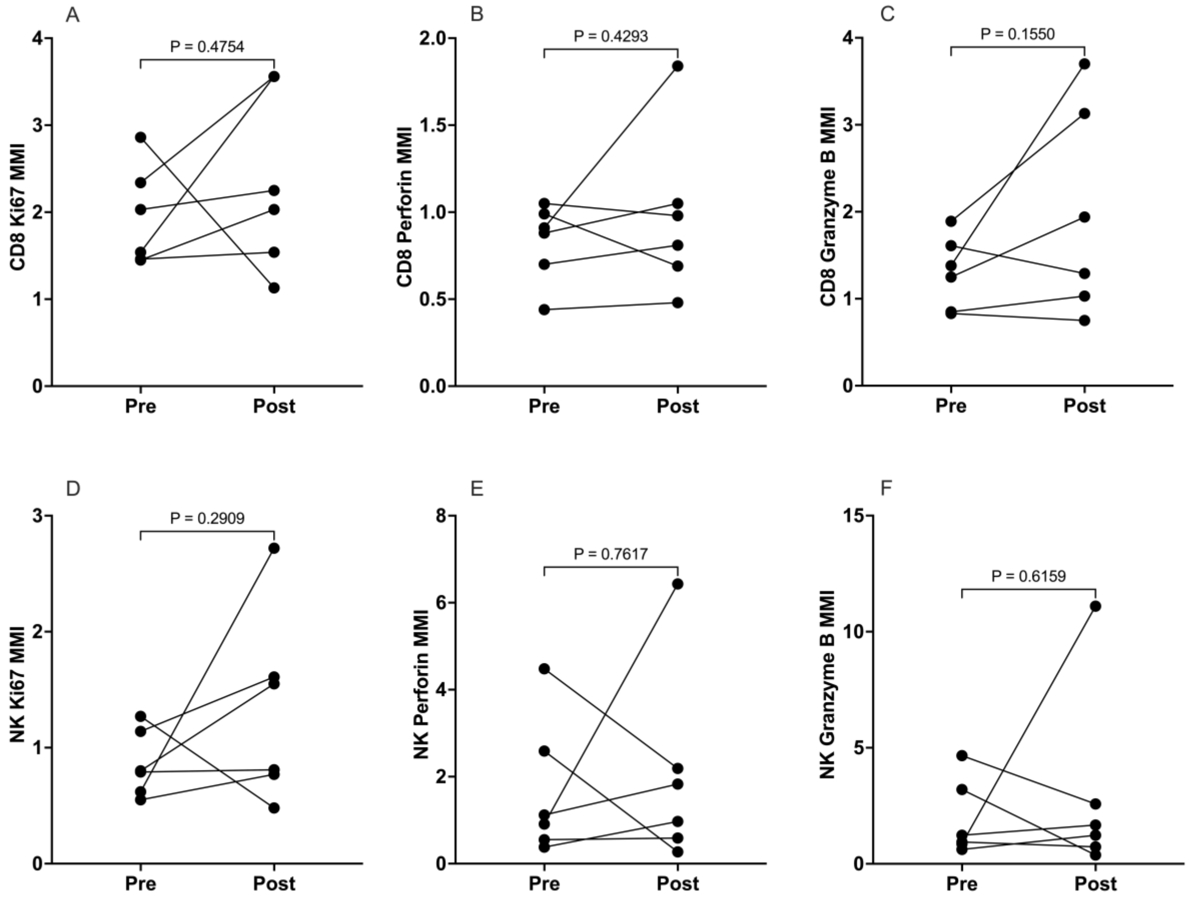

Supplemental Figure 3. Gating scheme for CyTOF data analyses. NK cells were gated by expression of CD56 and/or CD16 after exclusion of dead cells and monocytes, B cells, and T cells.

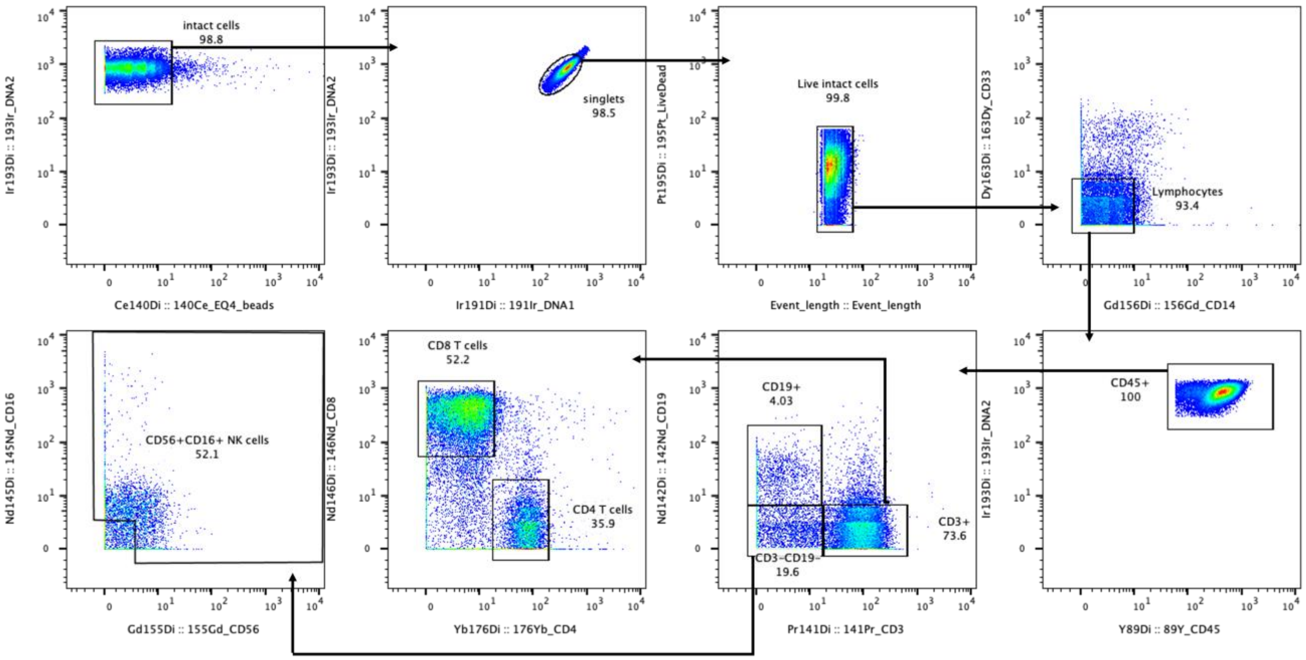

Supplemental Figure 4. Comparison of CyTOF staining vs. flow cytometry staining of NK cells. Various donor PBMCs were assessed for NK cells in the lymphocyte population by flow cytometry and CyTOF after live/dead and T cell exclusion. Matching PBMCs and the same antibody clones were used for both the flow cytometry and the CyTOF staining.

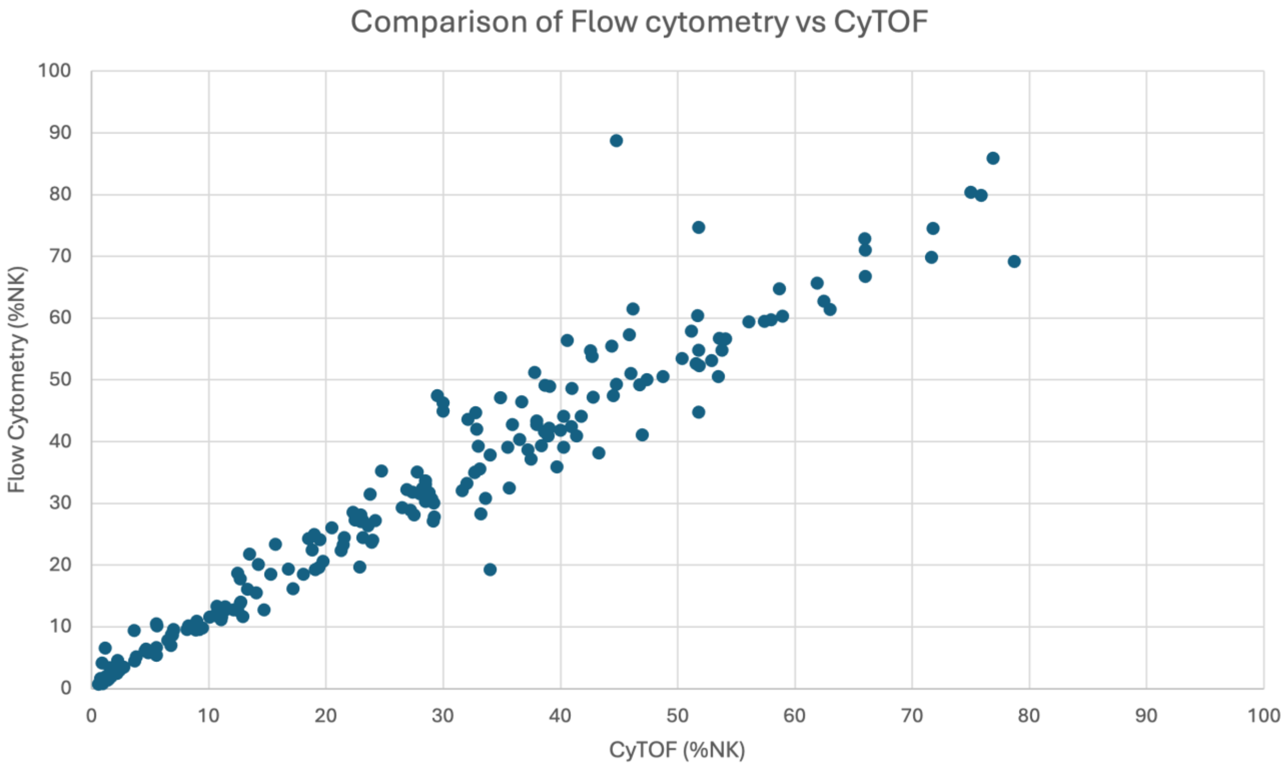

Supplemental Table 1. Clinical adverse events (AEs) data in study participants. The table shows clinical AEs identified in the National Institute of Health (NIH) Division of AIDS (DAIDS) Table for Grading the Severity of Adult and Pediatric AEs, corrected version 2.1 (July 2017), or AEs otherwise relevant to this study. All participants (10/10) had grade 1, 7/10 had grade 2, and 8/10 had grade 3 clinical AEs. There were no grade 4 AEs.

**Clinical adverse event**	**Grading[A]**			**No. affected (any grade)[B]**	**Relation to N-803**				**Relation to lymph node biopsy or colonoscopy**		
	**G1**	**G2**	**G3**		**Unrelated**	**Unlikely**	**Probable or possible**	**Definite**	**Unrelated**	**Probable or possible**	**Definite**
Injection site erythema/redness	8	5	16	10 (100%)				29	29		
Injection site induration/swelling	3	-	2	3 (30%)			2	3	5		
Injection site tenderness	1	-	-	1 (10%)				1	1		
Incision site pain[C]	5	-	-	3 (30%)	5						5
Incision site erythema[C]	1	-	-	1 (10%)	1						1
Lymphadenopathy[C,D]	12	-	-	6 (60%)			12		12		
Myalgia	16	-	-	9 (90%)	1		15		16		
Headache	16	-	-	9 (90%)	4	2	10		16		
Fatigue or malaise	14	-	-	8 (80%)	1	1	12		14		
Pain[E]	8	1	-	7 (70%)	5	1	3		7	2	
Fever	10	1	-	5 (50%)	1		10		11		
Night sweats[C]	7	-	-	3 (30%)			7		7		
Anorexia	7	-	-	5 (50%)	1	1	5		6	1	
Arthralgia	7	-	-	5 (50%)		1	6		7		
Nausea	6	-	-	5 (50%)	1	1	4		6		
Neurosensory alteration (numbness, paresthesia)	4	-	-	3 (30%)	1		3		3		1
Altered mental status (lethargy)	4	-	-	3 (30%)			4		4		
Vomiting	3	-	-	3 (30%)	1		2		2		1
Gastrointestinal bleeding	2	-	-	2 (20%)	2					1	1
Constipation	2	-	-	2 (20%)		2			2		
Rash	1	1	-	2 (20%)		1		1	2		
Dyspnea	2	-	-	1 (10%)	1		1		2		

Chills	1	-	-	1 (10%)			1	1	
Diarrhea	1	-	-	1 (10%)		1		1	
Depression	1	-	-	1 (10%)		1		1	
Odynophagia/tonsilliti s	1	-	-	1 (10%)	1			1	
Pain during colonoscopy[C]	1	-	-	1 (10%)	1				1
Other AE[F]	25	3	-	9 (90%)	13	11	4	28	

[A]The instances documented for each AE are shown by grade (G1, G2, and G3) as defined by the DAIDS AE Table.
[B]This is the AE prevalence of clinical AEs among study participants.
[C]These AEs are not listed in the DAIDS AE Table but are of relevance to our study. Per protocol, we defined lymphadenopathy grading as follows: grade 1 (increase in LN size to >5 cm by palpation), grade 2 (increase in LN size to >5 cm lasting 7–14 days or ultrasound evidence of LN necrosis), and grade 3 (increase in LN size to >5 cm lasting >14 days or adenitis diagnosed by computed tomography with fever >101.5°F or any other evidence of infection).
[D]Lymphadenopathy was reported in axillae, groin, and/or neck.
[E]Pain reported included arm pain, neck pain, back pain, testicle pain, chest pain, and abdominal pain.
[F]Other AEs included events not identified in the DAIDS AE Table. Other AEs were nasal or sinus congestion (3 events in 2 people), cough (3 events in 2 people), skin lesions/abrasions/wounds (3 events in 2 people), dry mouth (2 events in 1 person), COVID-19 (2 events in 2 people), fetid urine (2 events in 2 people), dark urine (1), greasy stool (1), dizziness (1), upper respiratory infection (1), muscle spasm (1), increased appetite (1), mycosis (1), increased thirst (1), gonorrhea (1), *Helicobacter pylori* infection (1), dental crown fracture (1), gingivitis (1), and tonsillolith (1). The 3 G2 AEs in this category were COVID-19, gonorrhea, and *H. pylori* infection, all of them not related to N-803 or study procedures.

Supplemental Table 2. Laboratory AEs data in study participants. The table shows laboratory AEs identified in the NIH DAIDS Table for Grading the Severity of Adult and Pediatric AEs, corrected version 2.1 (July 2017). All participants (10/10) had grade 1, 7/10 had grade 2, and 2/10 had grade 3 laboratory AEs. There were no grade 4 AEs.

**Laboratory adverse event**[A]	**Grading**[B]			**No. affected (any grade)**[C]
	**G1**	**G2**	**G3**	
Creatinine clearance (eGFR), low	NA	21	4	6 (60%)
Albumin, low<br>Normal range, 3.4–5.0 g/dL	7	-	-	5 (50%)
Glucose (non-fasting), high<br>Normal range, 70–99 mg/dL	7	2	-	4 (40%)
Creatinine, high<br>Normal range, 0.66–1.25 mg/dL	2	-	-	2 (20%)
ALT, high<br>Normal range, 0–70 U/L	2	-	-	2 (20%)
Absolute neutrophil count, low<br>Normal range, 1.6–8.3 10e3/μL	2	-	-	2 (20%)
Potassium, low<br>Normal range, 3.4–5.3 mmol/L	2	-	-	2 (20%)
Sodium, low<br>Normal range, 133–144 mmol/L	2	-	-	2 (20%)
Calcium, high<br>Normal range, 8.5–10.1 mg/dL	1	-	-	1 (10%)
Glucose (non-fasting), low<br>Normal range, 70–99 mg/dL	1	-	-	1 (10%)

[A]There were no laboratory AEs related to AST, alkaline phosphatase, total bilirubin, hemoglobin, white blood cells, platelets, QTc interval, or pulmonary function tests. Absolute lymphocyte counts are not assessed in PLHIV per DAIDS AE Table.

[B]The instances documented for each laboratory AE are shown by grade (G1, G2 and G3) as defined by the DAIDS AE Table.

[C]This is the AE prevalence of laboratory AEs among study participants.

eGFR, estimated glomerular filtration rate; NA, not applicable.

Supplemental Table 3. List of antibodies used for IHC and CyTOF analyses.

Target marker / antibody	Metal tag	Clone	Reference/Product ID/Catalog #
NKG2A (abcam)		EPR23737-127 (Rabbit)	ab260035
CD8 (Thermo)		SP16 (Rabbit)	MA514548
CD20 (Biocare)		L26 (Mouse)	CM004A
Mouse Secondary HRP (GBI)			D37-110
Rabbit Secondary HRP (GBI)			D39-110
CD3	141Pr	UCHT1	3141019B
CD19	142Nd	HiB19	3142001B
CD8a	146Nd	RPA-T8	3146001B
CD56 (NCAM)	155Gd	B159	3155008B
CD14	156Gd	HCD14	3156019B
Ki67	162Dy	B56	3162012B
CD33	163Dy	WM53	3163023B
Granzyme B	171Yb	GB11	3171002B
Perforin	175Lu	B-D48	3175004B
CD4	176Yb	RPA-T4	3176010B
CD45	89Y	Hi30	3089003B
CD16	145Nd	3G8	3145008B

Supplemental Methods. Selection criteria of the clinical trial.

Inclusion criteria

1. Male or female, age ≥ 18 and ≤ 65 years.
2. HIV-1 infection, documented by any licensed rapid HIV test or HIV enzyme or chemiluminescence immunoassay (E/CIA) test kit at any time prior to study entry and confirmed by a licensed Western blot or a second antibody test by a method other than the initial rapid HIV and/or E/CIA, or by HIV-1 antigen or plasma HIV-1 RNA viral load.
3. On continuous antiretroviral therapy for over 24 months without any interruptions of greater than 14 consecutive days, without plans to modify ART during the study period.
4. Screening plasma HIV RNA levels < 20 copies/mL and on at least 1 determination in past 12 months (isolated single values ≥ 20 but < 200 copies/mL will be allowed if they were preceded and followed by undetectable viral load determinations).
5. Screening CD4+ T cell count ≥ 350 cells/mm^3.
6. Ability to be off prednisone and other immunosuppressive drugs for at least 14 days before screen. Inhaled, nasal spray, intraarticular injections, and topical steroids are acceptable.
7. Acceptable blood pressure and heart rate parameters within normal limits (systolic = 88–140 mmHg; diastolic = 50–<90 mmHg; heart rate = 46–100 bpm). Treatment with antihypertensive medication is allowed. However, if someone is on a beta-blocker this must be switched to another class of medication as there is a theoretical risk for bradycardia if the participant were to experience cytokine release syndrome symptoms (which has not happened with this drug delivered s.c.).
8. Sexually active females of childbearing potential and males with partners of childbearing potential must agree to use effective contraception during study participation and for 1 month following the final study visit (4 months after final dose of study drug). Acceptable birth control for female participants of childbearing potential is defined as two of the following forms of contraception, one of which must be a barrier method:
    a. Condoms (male or female) with or without a spermicidal agent.
    b. Diaphragm or cervical cap with spermicide.
    c. Intrauterine device (IUD) with published data showing that expected failure rate is < 1% per year.
    d. Tubal ligation.
    e. Hormone-based contraceptive such as oral birth control pills.
9. Laboratory tests performed within 14 days of study enrollment must be a grade 0 or 1 as defined by the Division of AIDS Table for Grading the Severity of Adult and Pediatric Adverse Events, Corrected Version 2.1, with the following exceptions:
    a. Platelet count (≥ 150,000/mm^3).
    b. Hemoglobin > 12.5 g/dL for men and > 11.5 g/dL for women. It is not acceptable for patients to be transfused to meet this requirement. The use of Epogen is permitted.
    c. Estimated CrCl (eGFR) > 50 ml/min.
10. Voluntary written consent provided by the participant.

Exclusion criteria

1. Active or recent malignancy requiring systemic chemotherapy or surgery in the preceding 36 months or for whom such therapies are expected in the subsequent 12 months. Minor surgical removal of localized skin cancers (squamous cell carcinoma, basal cell carcinoma) are not exclusionary.

2. Chronic liver disease defined as Class B and C on the Child-Pugh chronic liver disease score.
3. Active and poorly controlled atherosclerotic cardiovascular disease (ASCVD), as defined by 2013 ACC/AHA guidelines, including a previous diagnosis of any of the following:
    a. acute myocardial infarction.
    b. acute coronary syndromes.
    c. stable or unstable angina.
    d. coronary or other arterial revascularization.
    e. stroke.
    f. transient ischemic attack (TIA).
    g. peripheral arterial disease presumed to be of atherosclerotic origin.
4. History of potential immune-mediated medical conditions requiring concomitant treatment with immunomodulatory drugs, and/or exposure to any immunomodulatory drug in the 30 days prior to screen (e.g., corticosteroid therapy equal to or exceeding a dose of 15 mg/day of prednisone for more than 10 days, IL-2, interferon, methotrexate, cancer chemotherapy). Use of inhaled, nasal steroid, or topical steroid lotions and creams is not exclusionary. Prior exposure to N-803 is not exclusionary if prior exposure occurred at least 6 months before screen.
5. Exposure to any experimental therapies within 90 days of study screen. Exposure to long-acting injectable ART is not exclusionary.
6. Latent TB infection or active TB disease prior to completing a standard regimen of anti-TB therapy that is defined as meeting PPD criteria for TB exposure or a positive QuantiFERON-TB Gold test (QFT) collected at screening.
7. Active fungal infection requiring systemic antifungal therapy.
8. Active herpes outbreak or varicella-zoster virus infection requiring episodic treatment.
9. Chronic active hepatitis B or C. For hepatitis B this will be defined as HBs antigen + and for hepatitis C this will be defined as HCV antibody + and HCV PCR +.
10. History and/or presence of any clinically significant disease or disorder, such as cardiovascular, pulmonary, renal, hepatic, neurological, gastrointestinal, and psychiatric/mental disease/disorder, which, in the opinion of the site principal investigator, may either put the participant at risk because of participation in the study, influence the results of the study or the participant's ability to be in the study.
11. Any degree of baseline QT/QTc interval prolongation (QTc interval > 450 ms in men and > 470 ms in women).
12. Any ischemic changes seen in the stress treadmill test administered per the discretion of the PI in order to assess any other ECG abnormalities.
13. Marked abnormalities in pulmonary function test (PFT).
14. Obesity if deemed exclusionary to conduct LN biopsy.
15. History or evidence of uncontrollable CNS disease such as dementia, demyelinating disease, Parkinson disease, or a CNS degenerative disease that, in the opinion of the site principal investigator, may either put the participant at risk because of participation in the study, influence the results of the study or the participant's ability to be in the study.
16. Prior organ allograft or allogeneic transplantation.
17. Planning or current pregnancy or breastfeeding.
18. Any clinically indicated vaccination (other than influenza or SARS-CoV-2) administered within 14 days of screen.
